# Langmuir–Blodgett Graphene-Based Films for Algal Biophotovoltaic Fuel Cells

**DOI:** 10.3390/nano12050840

**Published:** 2022-03-02

**Authors:** Vengadesh Periasamy, Muhammad Musoddiq Jaafar, Karthikeyan Chandrasekaran, Sara Talebi, Fong Lee Ng, Siew Moi Phang, Georgepeter Gnana kumar, Mitsumasa Iwamoto

**Affiliations:** 1 Low Dimensional Materials Research Centre (LDMRC), Department of Physics, Faculty of Science, University of Malaya, Kuala Lumpur 50603, Malaysia; sara@eprofilersolutions.com; 2 Institute of Ocean and Earth Sciences (IOES), University of Malaya, Kuala Lumpur 50603, Malaysia; sekarkarthik1603@um.edu.my (K.C.); fonglee_ng@yahoo.com (F.L.N.); 3 Institute of Microengineering and Nanoelectronics, Research Complex, Universiti Kebangsaan Malaysia, Bangi 43600, Malaysia; m.musoddiq@gmail.com; 4 International College of Semiconductor Technology, National Yang Ming Chiao Tung University, University Road, Hsinchu 30010, Taiwan; 5 Institute of Biological Sciences, Faculty of Science, University of Malaya, Kuala Lumpur 50603, Malaysia; 6 Faculty of Applied Sciences, UCSI University, Kuala Lumpur 56000, Malaysia; 7 Faculty of Engineering Technology & Built Environment, UCSI University, Kuala Lumpur 56000, Malaysia; kumarg2006@gmail.com (G.G.k.); iwamoto.m.ac@m.titech.ac.jp (M.I.); 8 Department of Physical Chemistry, School of Chemistry, Madurai Kamaraj University, Madurai 625021, Tamil Nadu, India; 9 Department of Electrical and Electronic Engineering, Tokyo Institute of Technology, 2-12-1, S3-33 O-okayama, Meguro-ku, Tokyo 152-8552, Japan

**Keywords:** algae biophotovoltaic fuel cell, Langmuir–Blodgett, graphene, graphene oxide, reduced graphene oxide, microalgae, biophotovoltaics, fuel cell

## Abstract

The prevalence of photosynthesis, as the major natural solar energy transduction mechanism or biophotovoltaics (BPV), has always intrigued mankind. Over the last decades, we have learned to extract this renewable energy through continuously improving solid-state semiconductive devices, such as the photovoltaic solar cell. Direct utilization of plant-based BPVs has, however, been almost impracticable so far. Nevertheless, the electrochemical platform of fuel cells (FCs) relying on redox potentials of algae suspensions or biofilms on functionalized anode materials has in recent years increasingly been demonstrated to produce clean or carbon-negative electrical power generators. Interestingly, these algal BPVs offer unparalleled advantages, including carbon sequestration, bioremediation and biomass harvesting, while producing electricity. The development of high performance and durable BPVs is dependent on upgraded anode materials with electrochemically dynamic nanostructures. However, the current challenges in the optimization of anode materials remain significant barriers towards the development of commercially viable technology. In this context, two-dimensional (2D) graphene-based carbonaceous material has widely been exploited in such FCs due to its flexible surface functionalization properties. Attempts to economically improve power outputs have, however, been futile owing to molecular scale disorders that limit efficient charge coupling for maximum power generation within the anodic films. Recently, Langmuir–Blodgett (LB) film has been substantiated as an efficacious film-forming technique to tackle the above limitations of algal BPVs; however, the aforesaid technology remains vastly untapped in BPVs. An in-depth electromechanistic view of the fabrication of LB films and their electron transference mechanisms is of huge significance for the scalability of BPVs. However, an inclusive review of LB films applicable to BPVs has yet to be undertaken, prohibiting futuristic applications. Consequently, we report an inclusive description of a contextual outline, functional principles, the LB film-formation mechanism, recent endeavors in developing LB films and acute encounters with prevailing BPV anode materials. Furthermore, the research and scale-up challenges relating to LB film-integrated BPVs are presented along with innovative perceptions of how to improve their practicability in scale-up processes.

## 1. Introduction

Booming energy demands and the exhaustion of toxic gases from conventional fuel sources compel research endeavors in the design and development of sustainable energy conversion devices with zero pollution. In this line, sustainable energy generation using BPV fuel cells (FCs) has currently attracted research and development endeavors owing to its advantageous characteristics, including carbon sequestration, bioremediation and biomass production [1,2]. Furthermore, algae-based BPVs are self-sustainable and do not require a supply of external organic carbon sources, enhancing their status with respect to the other conventional microbial FCs [3,4]. Algal BPV FCs utilize photosynthetic microalgae or cyanobacteria, requiring sunlight (energy) and water (source of electrons) for the generation of bioelectricity [5]. This carbon-neutral/negative energy conversion [6] involves the generation of bioelectricity via two different phenomena under light and dark regimes. The primary photosynthesis process involves photocurrent production arising from the photosynthetic electron transfer chain (PETC) upon light irradiation. This serves as a stimulus for the oxidation of water molecules, generating protons (H^+^), electrons and oxygen [7], while the breakdown of stored carbohydrates with the use of photosynthetic by-products is responsible for the production of a dark current.

The power generation efficacy of algae-based BPVs is significantly influenced by the anode material and its functionalization. Graphene (periodic arrays of densely packed carbon atoms in a 2D honeycomb structure) and its derivatives of graphene oxide (GO) and reduced graphene oxide (rGO) have been considered as excellent anode materials owing to their constructive characteristics, including lower thickness, high mechanical stability, optical transparency, etc. Specifically, the graphene-based thin films demonstrate a higher degree of geometric order and a large surface area, resulting in the desirable electronic properties that could be utilized in state-of-the-art electronics [8]. Derivatives of graphene, meanwhile, such as GO [9,10,11,12,13], rGO [14,15,16] and graphene sheets (GS) [17,18], prepared in the form of thin films or sheets have shown significantly improved structural and surface dynamics. These include lower density, high porosity, surface roughness, larger specific and relative surface area, hydrophobicity, excellent mechanical strength and electrochemical performance for high-performance electronics [19,20,21,22,23]. With relatively higher sensitivity to local changes in terms of conductivity when compared to bulk material preparation, such as the conventional catalyst-loaded electrodes, graphene-based thin films prove beneficial for sensing purposes, absorbing or desorbing molecules on their surfaces and other properties.

Graphene-based thin films have therefore found various applications in electronics, photonics, energy generation and storage and in biology and medicine [24,25,26,27,28,29]. In this context, various thin film-forming strategies, including template-directed assembly or chemical vapor deposition, self-assembly, electrochemical synthesis and centrifugal evaporation-induced methods have been developed. However, these methods are generally tedious and lengthy, involving complicated chemical processes at high pressures and temperatures and/or requiring hazardous gases, contributing significantly to higher production cost [13,17,30]. Interestingly, the Langmuir–Blodgett (LB) technique has been demonstrated as an effective yet straightforward method of manipulation at the molecular level for the fabrication of mono- or multilayered thin films at room temperature.

The LB method involves suspending amphiphilic molecules in a liquid subphase, generally deionized water, which spontaneously spread to form a monolayer or Langmuir film. With the use of moving barriers, these floating molecules are further compressed to form a solid-state monolayer film that can be transferred onto a solid substrate with the aid of a dipping process. Traditionally used to assemble homogenous organic and inorganic 2D thin films, structures formed using the LB method exhibit exceptionally higher degrees of geometric order, resulting in superior electronic properties, which are not achievable using other methods [31,32,33]. Using an unconventional approach, researchers have also demonstrated deposition of three-dimensional (3D) rGO sheets instead of the conventional 2D structures for potential application as anode materials [34]. The 3D topography increased the surface area significantly and therefore enhanced biocompatibility or adhesion, which provided the optimum contact for excellent electrical coupling of biologically active materials, such as the microalgae for BPV FC applications.

Fabrication of graphene-based anodes as biocompatible materials, especially using the LB technique, may therefore assist the development of algal BPV platforms for improved power outputs. Although adequate literature is available for thin films generated by the LB technique for FCs, there is not as yet an approachable guide that addresses and tackles the chief issues hampering the evolution of practical BPVs. Therefore, the current review aims to provide a compact yet descriptive discussion pertaining to the most recent achievements in the research and development of algal BPV FCs based on LB-assisted graphene-based anode materials.

## 2. Requirements of Anode Materials for BPV 

**Figure 1:**
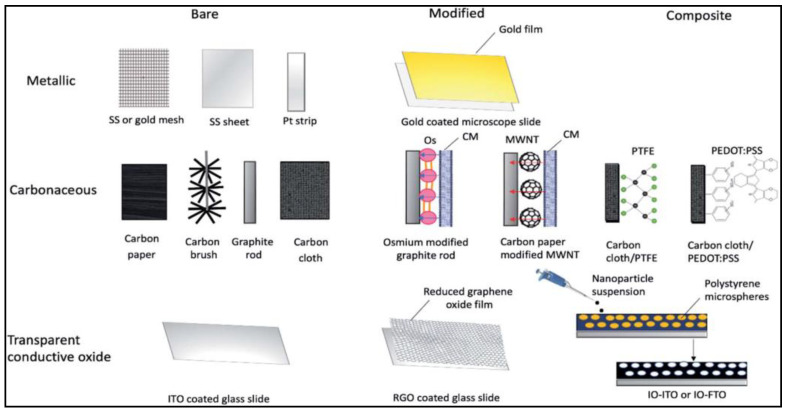
Summary of various electrode configurations used in biophotovoltaic systems. Abbreviation: SS, stainless steel; Pt, platinum; Os, osmium; CM, cytoplasmic membrane; MWNT, multiwalled nanotube; PTFE, polytetrafluoroethylene; PEDOT:PSS, poly(3,4-ethylenedioxythiophene):polystyrene sulfonate; ITO, indium tin oxide; IO-ITO, inverse opal indium tin oxide; IO-FTO, inverse opal fluorine doped tin oxide. Reprinted with permission from Sustainable Energy & Fuels, copyright 2021, Royal Society of Chemistry [1].

In general, certain properties are required when it comes to anodes for algae-based and microbial fuel cells (MFCs), prioritized by good biocompatibility, large surface area, high chemical, thermal and mechanical stability and high conductivity. So far, major improvements have been achieved by means of modifying the available anodes applied in FCs, especially in terms of power generation. This remains a challenge, though, since the various methods employed in anode fabrication do not have or have only limited flexibility as is necessary to allow a wide range of modifications for optimizing the anode in order to cater for the many different types of microalgae utilized for BPVs, bioremediation, etc. A good overview of the recent advances in anodes, especially for MFCs, can be found in the following review article by Yaqoob and co-workers [35]. Figure 1 below, lists different types of carbon-based, metallic and transparent conductive oxide anode materials and electrodes/anodes currently being used in FCs and MFCs [1].

Conventional anodes are generally optimized for mainstream FC applications which relate to non-biological photovoltaic devices. While great progress in this aspect has contributed towards improved anode performances, specific functionalization (usually not observed in standard FC research) must be adhered to when it comes to BPVs, such as those involving microalgae. Most important is the adhesion and/or biocompatibility of the anodes towards algae, achieved by means of optimizing anode porosity and control of functional groups. Understanding the properties of the selected algal species, such as their size, enables the fabrication of anodes with corresponding pore sizes and functional groups. In a previous work, the LB technique was employed successfully to allow flexibility in controlling pore sizes which accounted for the improved charge transferability by means of high surface area and algae adhesion [36]. The LB technique was also demonstrated to exclusively allow monolayer-by-monolayer deposition to fabricate highly uniform anode films, the level of which is not possibly controllable using other techniques.

## 3. Fundamentals of the LB Technique and Its Applications for Graphene-Based Films 

The LB technique is a popular method due to its innate ability to produce large areas of highly uniform thin films, in addition to allowing the re-engineering and manipulation of structures on nano- and microscales [11,34,37] to produce films of different density and packing and with different arrangements of physical properties [38]. In addition, the LB technique does not involve extreme working conditions, such as high temperature and pressure or release of harmful and toxic chemicals, making it an inexpensive and environmentally friendly technique.

GO is generally preferred for producing LB films compared to graphene due to its natural amphiphilic properties [39]. The hydrophobic domain of GO is attributed to the π–π conjugated sp^2^ carbon, while the O group binding with the basal plane confers hydrophilic properties [40]. GO has low diffusivity in water, which is attributed to the ionizable edge of the -COOH group. It is in this regard that GO has been considered as an amphiphile with a largely hydrophobic basal plane and hydrophilic edge. Some amounts of GO do dissolve into the subphase; however, larger sheets typically float on the water surface while smaller ones might sink due to the increase in hydrophilicity, as reported by J. Kim et al. [39]. This amphiphilic property results in the formation of a stable water-insoluble monolayer, a prerequisite for a material to be used in LB assembly. A further reduction process yields rGO, which effectively reduces the hydrophilicity of the material. To obtain 2D rGO films, the synthesized rGO should be diluted with methanol, a polar alcohol, since rGO has the tendency to collapse and form 3D structures in the presence of non-polar solvents [41]. 

**Figure 2:**
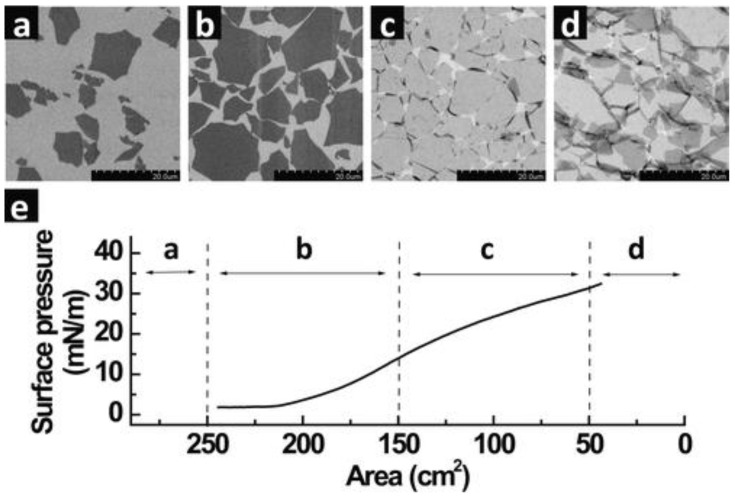
The LB assembly of single monolayers of GO on top of a silicon wafer with different regions of compression area: (**a**) dilute monolayers of isolated GO sheets, (**b**) close-packed arrangement, (**c**) over-packed monolayers with a folded sheet at interconnecting edges and (**d**) over-packed monolayers with folded and partially overlapped GO sheets interlocking with each other. (**e**) Isothermal surface pressure versus subphase (water) area plotted for the corresponding regions of (**a**–**d**). Scale bar is 20 µm. Reprinted with permission from the Journal of the American Chemical Society, copyright 2009, American Chemical Society [37].

An isotherm graph, which is typically a surface pressure versus area profile, generated upon compression of the GO monolayer on top of the water subphase is monitored using a tensiometer (Figure 2). The slope or the gradient of the isotherm defines the characteristics of the monolayer such that an increasing gradient demonstrates a state of increasingly reactive amphiphilic monolayer elements, while a decreasing value represents a state of collapse in which the elements leave the air/water interface. Barrier speed is set at the lowest possible speed, commonly around 7–10 mm/min or lower, to enable homogeneous and uniform monolayer compression. As the barriers start to compress the monolayer, a region of gaseous state can be observed (a) indicating the formation of individually isolated GO sheets as a result of well-dispersed sheets on the water subphase. At this stage, the surface pressure remains constant. Even though the region is a coexistence between isolated GO sheets and some of the sheets interacting with each other, the phase defined is merely the representation or the association of the surface pressure versus area. The physical properties of liquid and gases at microscopic dimensions are isotropic by narure, therefore they are not direction-dependent. Liquids and gases are therefore similar under certain conditions (in the case of GO spreading on a water surface) making it non-trivial to differentiate between these two states. As such, the 2D monolayer phase is generally defined as the condition in which molecules spread far apart on the surface exert significantly smaller forces on one another [31]. Upon continuous compression, the GO sheets come into contact with each other (b), increasing the surface pressure significantly as a result of the electrostatic repulsion of the individual GO sheets upon achieving a close-packed arrangement. Further compression of the barrier causes the surface pressure to continuously rise, albeit the gradient is lower (c) than the previous region in (b). At this stage, the monolayer goes beyond a close-packed arrangement and folding of the GO sheet appears at the interconnected edges. 

The monolayer is inclined to disrupt into multilayers around the edges due to the flexibility of the soft edges of the GO sheet. However, the central area of the GO sheet in general remains flat without much wrinkling or folding. The folding effect at the edge of the GO may be described by the effect of the slightly lower gradient of the isotherm graph representing the region in (c). Continuous compression finally pushes the LB film to breaking point at which the GO sheets start to buckle, wrinkle, overlap and/or partially overlap [42]. Smaller GO sheets seem to completely crumble while larger sheets maintain flatness in the center. This is due to the edge-to-edge interactions of the GO sheets on the water subphase during the compression process. The center of the GO sheet maintains flatness, while the edges buckle, wrinkle, overlap and/or partially overlap, as mentioned earlier due to the repulsion at the edges. The GO sheet at the center of the subphase will, however, remain flat until it reaches the point where no more free space is left upon further continuous compression [37].

The final step in the formation of 2D graphene-based film is the transfer and deposition of the GO layer or film onto a solid substrate using the dipping process. This method, however, proves challenging for producing thin films of large area [43,44]. Hence, numerous methods have been developed, especially to improve the deposition of rGO and GO films onto solid substrates. These includes using hydrophobic substrates [45], a roll-to-roll deposition process [46], controlled edge-to-edge assembly of floating films [43] and guided transfer by adjusting the substrate to a shallow angle [44]. Cote and co-workers determined the optimum gap size of the GO sheet at each stage of arrangement by dipping solid substrates at various surface pressures representing close-packed to nearly over-packed arrangements [37]. The resulting surface morphology of the GO layers was then analyzed using atomic force microscopy (AFM). Variation in gap sizes was observed for each arrangement of GO sheets, even at over-packed stages where nanogaps were observed due to electrostatic repulsion between the sheets.

## 4. Recent Research Efforts

### 4.1. Graphene-Based LB Films for Fuel Cell Applications 

Traditional and manipulated LB deposition methods have been reported to yield high-quality and large 2D rGO or GO thin films. The traditional method involves vertical dipping of the substrate through a water subphase during the close-packing arrangement of rGO sheets [37]. For efficient material adhesion and for effective draining of water, a vertical dipping process could be carried out at a very low speed to increase adhesion time, as rGO will not attach well to the substrate at high dipping speeds [34]. The resulting 2D rGO thin film is then air-dried at room temperature or kept in an oven to enhance the drying process [18].

The traditional method can be manipulated in several ways to deposit large-area 2D GO films. Xu and co-workers demonstrated a controlled edge-to-edge assembly of a floating 2D GO monolayer with the ability to yield large-area films [43]. The method includes an improved spreading of solvent using ethanol/1, 2-dichloroethane with a volume ratio of 1:13 instead of the traditional methanol/water (ratio 5:1). As a result, the transfer efficiency increased by five-fold and a large accumulation of GO monolayer was achieved. 

The mechanism of controlled edge-to-edge assembly contributes to barrier-free densification, the aggregation mechanism and spreading-induced film. The barrier-free densification of the LB film is accomplished by using repetitive dripping deposition of 0.025 mg/mL GO dispersion at the air–water interface. This method proves to be nearly 100% efficient in transferring a GO monolayer onto a large-area substrate at the air–water interface and is attributed to the immersion forces that occur while the spreading solvent evaporates. The aggregation mechanism is carried out by depositing GO dispersion at a low pH onto a highly ionic subphase, which results in the shielding of repulsive electrostatic forces between individual GO sheets. Spreading-induced film densification is achieved by spreading solvent at a suitable distance-dependent force from floating GO films. Compression of barriers will then effectively assist to form a densely tiled film. The distance-dependent force is transmitted throughout the LB trough dissimilarly depending on whether the interaction between the sheets is governed by repulsive or attractive forces. 

**Figure 3:**
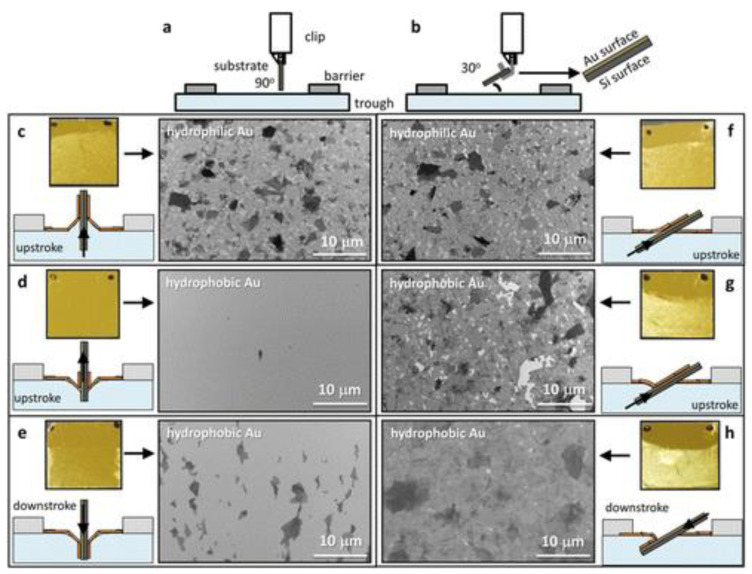
A comparison of conventional (**a**) 90° and (**b**) 30° dipping mechanisms of upstroke and downstroke of different hydrophobic and hydrophilic substrates. The SEM images show the comparison of the GO film formations in the upstroke (**c**,**f**) hydrophilic and (**d**,**g**) hydrophobic substrates of Au at both 90° and 30°. (**e**,**h**) SEM film formations as a result of downstroke using a hydrophobic Au substrate. Reprinted with permission from the Journal of the American Chemical Society, copyright 2015, American Chemical Society [44].

Using this mechanism, a continuous deposition of 2D GO film onto a large-area substrate for coating and patterning using roll-to-roll deposition was achieved [43]. In another report, the quality of the 2D films was significantly improved by manipulating the monolayer transfer process by adjusting the solid substrate at a slight angle during the dipping [44]. This observed improvement was due to the beneficiation of shear modulus causing GO to behave like a 2D substrate on the water subphase as a result of the strong interaction of each individual GO sheet at the air–water interface. Furthermore, the implementation of a shallow-angled substrate during the dipping process projects a minimum tensile stress during the insertion and extraction of the substrate (Figure 3). Poor GO adhesion onto hydrophobic surfaces resulting in low-quality film formation or coating when using a conventional vertical dipping method can be overcome by adjusting the dipping angle of the substrate to 30°, which creates a desirable meniscus radius of curvature.

Three-dimensional graphene-based materials, meanwhile, are of particular interest due to their intrinsic properties similar to 2D graphene sheets while they also have the added benefits of increased surface areas combining meso-, macro- and micropores. The meso- and microporosity display a highly specific area and macroporosity significantly improves the catalytic performance at the surface region [47]. These 3D porous structures coupled with the intrinsic properties of graphene impart high specific surface areas, durable mechanical strength and fast mass and electron transport kinetics. In line with this, several non-LB techniques have been developed to fabricate 3D structures. These include step-by-step assembly methods using graphene, GO or rGO sheets and direct synthesis from a carbon source (methane, ethanol or sugar) [34,48,49]. 

**Figure 4:**
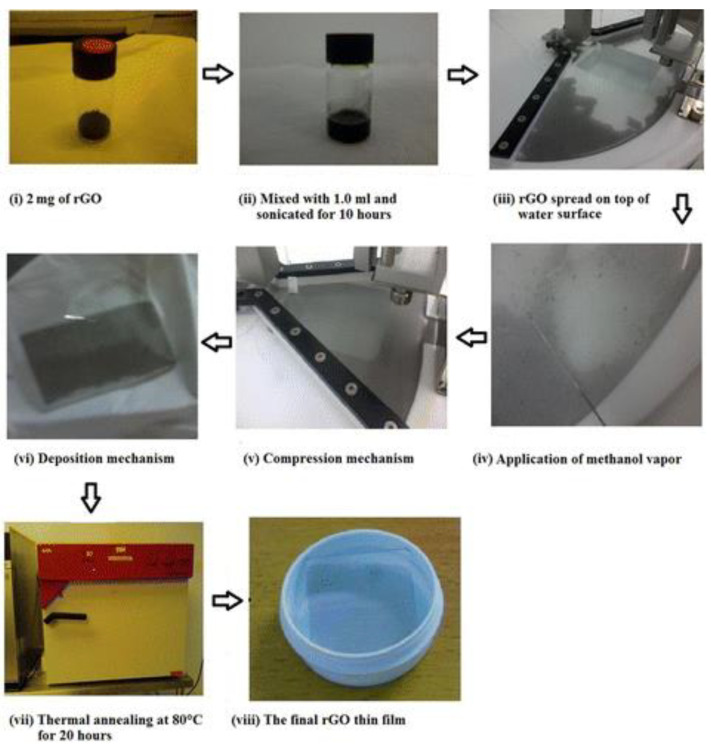
Flowchart showing the preparation of rGO LB film. To distribute the rGO flakes evenly, methanol vapor from a methanol-soaked tissue was applied to gently force the clouded regions apart to significantly improve the film uniformity. Reprinted with permission from ACS Publications, copyright 2015, American Chemical Society [34].

In a recent report, an unconventional approach was employed using the LB method for preparing 3D rGO films [34]. While dipping is conventionally carried out at the solid-state target pressure for preparing 2D LB film (Figure 4), Jaafar and co-workers carried out the deposition beyond the target pressure up to the breaking point. Beyond the breaking point, collapse or breakdown in the arrangement of the monolayer results in the formation of multilayers, providing an overall 3D morphology. Wrinkling effects are predominant while electrostatic repulsion between the 2D layers increases the average thickness [42]. Moreover, this may also increase the average roughness when the sheets crumble and wrinkle unevenly at different areas. Repeated transfer or layer-by-layer deposition further improves the 3D structures.

Annealing at 60 °C between each consecutive deposition of layers helps to create higher surface roughness and improves the porosity of the 3D structures. It was reported that at the sixth deposition layer of rGO, complex interconnected micrometer-scale porous structures were achieved which enabled enhanced integration and encapsulation of microalgae within BPV FCs for increased power output [36]. At higher deposition cycles, the roughness and pore size were increased, thus adjustment of the deposition cycles could be a useful way of achieving various roughness and pore sizes of 3D structures. These tunable properties may play important roles in cellular interactions by influencing cell behavior in biological microenvironments, such as in the rGO biofilms [50,51,52,53,54,55]. Compared to 2D graphene-based films, 3D structures provide excellent cellular communication, transportation of oxygen and nutrients, removal of waste and improved cellular metabolism. Furthermore, high porosity allows significant improvement in the adhesion of biomaterials and, in the case of microalgae, allows enhanced growth. As such, the LB technique provides a practical solution for controlling and optimizing surface roughness and pore size with respect to its intended applications.

### 4.2. Progress in Algae-Based Biophotovolatics 

Bombelli and co-workers used two types of photosynthetic materials: a whole cell of *Synechocystis* sp. and a sub-cellular photosynthetic membrane (the thylakoid from *Spinaciaoleracea* L.) for comparison of relative lifespan and power output [7]. The sub-cellular photosynthetic membrane produces a high-power output (4.56 nW (nmolChl)^−1^) compared to *Synechocystis* sp. (4.10 nW (nmolChl)^−1^). Regarding *Synechocystis* are cyanobacteria that produce microbial nanowires with pili-like structures (4.5–7 nm in diameter, around 2–10 μm long), Sure 2015 reported that the amino acid sequence of these structures was composed of aromatic amino acids, which explained its conductive characteristics [56]. In another work, successful growth of *Synechocystis* sp. on an alloy anode in a microfluidic BPV device was achieved, which generated a power output of 294 mW m^−2^ [57]. 

Bateson and co-workers, meanwhile, fabricated a bio-bottle-voltaic (BBV) device built from recycled plastic bottles and a recycled aluminium (Al) anode using green alga, *Chlorella sorokiniana* [58]. The system was able to deliver up to 2000 mC bottle^−1^·day^−1^ for 35 days. Prior to this, Nishio and co-workers had investigated the syntrophic relationship between green algae and heterotrophic bacteria in a photosynthetic microbial FC [59]. In this work, *Chlamydomonas reinhardtii* and *Geobactersulfur reducensi* growing on an anode (graphite felt) and carbon cloth coated with platinum (0.5 mg m^−2^) as the cathode achieved a power output of 9.2 mW m^−2^. The authors concluded that excretion of organic compounds by the algae resulted in the bottleneck in the electricity conversion process. 

The power outputs generated by algal BPV FCs are in general lower than conventional FCs. Accordingly, exogenous mediators have been used to counter the low power output by facilitating the electron transfer from the algae to the anode and reduce intrinsic metabolic losses and intracellular competition for energy resources [7,57,60]. However, the presence of such mediators imposes further limitations to efficiency in large scale applications since they would have to be separated and recycled due to toxicity and cost. To overcome this issue, algae biofilms were formed directly on the anode. Increase in power generation was observed due to the direct contact between the cells and electrode as a result of reduced internal potential losses [6]. Bradley and co-workers meanwhile recommended using whole algae cells that could efficiently transfer electrons to the anode. This is because microalgae are simple organisms that have the ability to self-assemble and self-repair [61].

**Figure 5:**
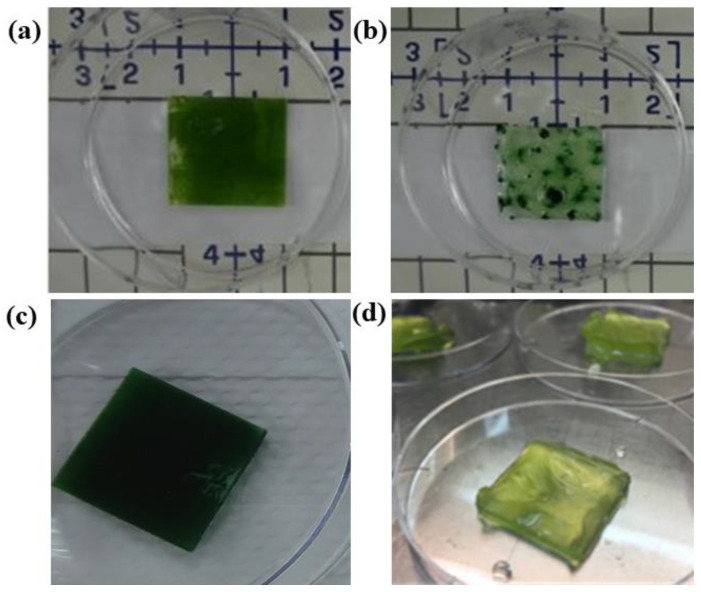
Formation of algal biofilms on anode materials. (**a**) *Chlorella* UMACC 313 on an ITO-coated glass anode, (**b**) *Spirulina* UMACC 159 on an ITO-coated glass anode, (**c**) *Synechococcus* UMACC 105 on an rGO-coated glass anode and (**d**) *Chlorella* UMACC 313 immobilized in alginate on top of the ITO coated glass anode. Photos are the properties of the authors of the paper.

BPV devices comprising algal biofilms on anodes were found to produce a higher power density compared to algae suspension on anodes [62]. Four algal strains, *Chlorella* UMACC 051 (0.112 mWm^−2^), *Spirulina* UMACC 159 (0.121 mWm^−2^), *Chlorella* UMACC 313 (0.124 mWm^−2^) and *Synechococcus elongates* UMACC 105 (0.313 mWm^−2^), generated the highest power densities among a number of species studied. In a work carried out in our laboratory, photosynthetically active algae immobilized in alginate gel were used to form an alginate–algal biofilm on an ITO anode (Figure 5). A peak power output of 0.29 mWm^−2^ representing an increase of 18% compared to a cell suspension culture BPV device was obtained. The suspension was then spread onto an ITO anode and allowed to settle under gravity; sterile CaCl_2_ (0.1 M) was used for the gelation process. *Chlorella* cells were embedded into the alginate matrix where compact colonies form upon cell division. This close-packed arrangement of the cells and its direct contact with the ITO anode reduced the liquid-phase mass transfer resistance, resulting in improved electron transportation [63]. 

## 5. Electrochemical Studies 

In terms of electrochemical properties, LB films of conjugated materials have been considered to be of prime interest in virtue of providing a mixed valence state of oxidation/reduction. Such materials demonstrate increased electrical conductivity owing to a partially oxidized or reduced state. Generally, well-ordered LB films demonstrate slow electrochemical redox reactions and electroactivity compared to non-well-ordered multilayer assemblies because LB films with small mean molecular areas favor diffusion-controlled voltammetry. The hindered electrochemical activity for multilayer LB films may have originated as a result of the formation of empty spaces between the hydrophobic chains due to surface tension effects. From a theoretical point of view, LB films attain vectorial electron transfer with an electron diffusion coefficient value of two orders of magnitude in comparison with multilayer and heterolayer films. The organized mono- and multilayers of LB films are therefore more importance for gaining insights into the interfacial electron transfer reactions [64]. The LB method therefore contributed an indispensable role in various electrochemical applications, including catalytic [65], electrochromic [66], electronic [67,68], photonic [68] and biosensing [69] devices.

Few studies in the literature have reported the electrochemical properties of LB films in biological FCs and only recently has there been some interest in this aspect. Jaffar and co-workers investigated the electrical properties of 3D rGO thin films integrated within a BPV FC and their interfacial electrochemical properties [34]. It was reported that the uniformly distributed pores present in a 3D rGO film improved the diffusion of ions in solution to all available space within the material. These pores not only increase mass transportation of ions in solution but also enhance the amount of charge the material can hold. Interestingly, the pores and roughness of the 3D rGO-based bio-electrode significantly improved the adhesion or immobilization of biocatalysts in biofuel cells. The improved adhesion significantly increased the amount of substrate that could be effectively consumed by the biocatalyst upon mass transportation. This bio-electrode demonstrated good biocompatibility with an increased current efficiency of 120% under the light cycle in comparison to conventional ITO-based BPV FCs. The LB assembly method also allows practical optimization of porosity from nanometer to micrometer scales upon layer-by-layer deposition, which will be useful as a favorable bio-electrode fabrication method for biosensors, biofuel cells and low-temperature capacitors. 

In another work, Ibrahim and co-workers utilized the LB method for the deposition of rGO films prior to plasma treatment by varying the plasma power [70]. As a result of the treatment, the wettability or hydrophilicity of the rGO film surface was improved. When a biofilm of *Chlorella* sp. was grown on the treated rGO anode (100 W plasma power), improved power density of 6.94 × 10^5^ W/m^2^ was obtained. A maximum surface roughness of 0.226 µm was achieved at 140 W of plasma power. Plasma treatment increased the adhesion of LB rGO layers on the glass substrate allowing substantially better biocompatibility with the microalgae, as evidenced by the cyclic voltammetry and chronoamperometric studies. The plasma-treated rGO anodes exhibited higher conductivity, porosity and surface roughness, which are the main contributing factors for BPV efficiency. The glass/rGO showed a prompt oxidation peak, observed at 0.87 V vs. Ag/AgCl, along with an improved oxidation current, which guarantees the presence of electroactive species/proteins located in the surface of *Chlorella* sp. under illumination. The increased anodic oxidation current under light conditions may have been established by the breakdown of intracellular metabolites and/or electron transfer to the photosynthetic chain by virtue of photosynthetic water splitting. Ng and co-workers meanwhile reported that an rGO anode in an algal BPV platform improved maximum power efficiency (0.141 mW/m^2^) against standard ITO anodes due to its intrinsic electrical properties and biological compatibility for microalgae [62]. 

Table 1 summarizes the major outputs of the works discussed above and some other recent works utilizing GO or rGO for the extraction of electrons in electrochemical applications. 

## 6. Conclusions

It could be understood that currently photosynthetic-based FCs significantly lags behind conventional FCs in terms of power generation. There are, however, advantages exclusive to BPV-based FCs, the most important of which is their carbon-negative nature. During BPV operation, electrons are produced through the photolysis of water molecules during photosynthesis and extracted by means of an external circuit for power generation. The liberated protons or the hydrogen atoms are later used to fix carbon atoms from carbon dioxide (CO_2_) to produce carbohydrates which effectively remove carbon from the environment. No harmful by-products are produced; further photosynthesis-based FCs release oxygen gas (O_2_) upon the photolysis of water molecules, contributing to healthy environmental oxygen level in terms of O_2_:CO_2_ ratios. BPVs have also been utilized successfully for bioremediation applications, with researchers developing algae-based BPV platforms for treating palm oil mill effluent (POME), wastewater and other substances. As such, the development of BPV-based FCs offers a comprehensive approach to renewable power generation superior even to solar cell technology, which validates continuous concentrated efforts. Therefore, the exciting prospects of producing clean green energy and/or biomass production while integrating bioremediation and wastewater treatment processes in a single platform is of immense interest in terms of global energy demands and climate change crisis.

## Figures and Tables

**Table 1:** Comparison of maximum current and power efficiency for different types of FCs.

Electrode	Catalyst	Fabrication Method	Types of FCs	Maximum Current/ Power Efficiency	Reference
ITO-PEN ^a^ ITO-PEN ^a^	TM ^b^/GO ^c^ TM ^b^	Electro-spraying	Photosynthetic electrons from plant	3.92 μA/cm^2^ 0.41 μA/cm^2^	[71]
GC ^d^	TM ^b^/rGO ^e^	Electro-reduction and electro-deposition	Photobioelectrochemical FCs	5.24 µA/cm^2^	[72]
ITO ^f^	GO ^c^-TM ^b^ rGO ^e^-TM ^b^	Drop-casting	Photoelectrochemical	21.37 µA/cm^2^ 18.95 µA/cm^2^	[73]
CC ^g^	TiO_2_/rGO ^e^	Electro-spinning	BPV	34.66 mW/m^2^	[74]
Glass	rGO ^e^ film	LB method	Biological	-	[34]
ITO ^f^	rGO ^e^ film	LB method	BPV	6.94 × 10^5^ W/m^2^	[70]
ITO ^f^	rGO ^e^ film	LB method	BPV	0.14 mW/m^2^	[62]

^a^ Indium tin oxide-coated polyethylene naphthalate; ^b^ thylakoid membrane; ^c^ grapheneoxide; ^d^ glassy carbon rotating disk electrodes; ^e^ reduced graphene oxide; ^f^ indium tin oxide; ^g^ carbon cloth.

## Data Availability

The data presented in this study are available on request from the corresponding author.
